# Heat-Induced Acceleration of Pozzolanic Reaction Under Restrained Conditions and Consequent Structural Modification

**DOI:** 10.3390/ma13132950

**Published:** 2020-07-01

**Authors:** Nankyoung Lee, Yeonung Jeong, Hyunuk Kang, Juhyuk Moon

**Affiliations:** 1 Department of Civil and Environmental Engineering, Seoul National University, 1 Gwanak-ro, Gwanak-gu, Seoul 08826, Korea; nankyoung@snu.ac.kr (N.L.); kanghu93@snu.ac.kr (H.K.); 2 Construction Technology Research Center, Korea Conformity Laboratories (KCL), 199 Gasan digital 1-ro, Geumcheon-gu, Seoul 08503, Korea; yeonungjeong@kcl.re.kr; 3 Institute of Construction and Environmental Engineering, Seoul National University, 1 Gwanak-ro, Gwanak-gu, Seoul 08826, Korea

**Keywords:** cement hydration, pozzolanic reaction, small-angle X-ray scattering, nuclear magnetic resonance

## Abstract

This study investigated the heat-induced acceleration of cement hydration and pozzolanic reaction focusing on mechanical performance and structural modification at the meso- and micro-scale. The pozzolanic reaction was implemented by substituting 20 wt.% of cement with silica fume, considered the typical dosage of silica fume in ultra-high performance concrete. By actively consuming a limited amount of water and outer-formed portlandite on the unreacted cement grains, it was confirmed that high-temperature curing greatly enhances the pozzolanic reaction when compared with cement hydration under the same environment. The rate of strength development from the dual reactions of cement hydration and pozzolanic reaction was increased. After the high-temperature curing, further strength development was negligible because of the limited space availability and preconsumption of water under a low water-to-cement environment. Since the pozzolanic reaction does not directly require the anhydrous cement, the reaction can be more easily accelerated under restrained conditions because it does not heavily rely on the diffusion of the limited amount of water. Therefore, it significantly increases the mean chain length of the C–S–H, the size of C–S–H globules with a higher surface fractal dimension. This finding will be helpful in understanding the complicated hydration mechanism of high-strength concrete or ultra-high performance concrete, which has a very low water-to-cement ratio.

## 1. Introduction

Cement undergoes a hydration reaction with water. If a sufficient amount of water is provided, the cement can completely react with the water to form cement hydrates, but if there is not enough water, numerous unhydrated particles remain in the hardened matrix. As a result, the amount of cement hydrates produced is generally related to the total porosity as well as the development of material properties, including the compressive strength. However, ultra-high performance concrete (UHPC) has a mixed proportion with a very low water-to-cement (w/c) ratio, ultimately coming with a large number of unhydrated cement particles [1,2,3]. Surprisingly, UHPC expresses high compressive strength—above 150 MPa—despite the large amount of anhydrous cement particles [1,2,4]. To explain the high strength of UHPC or high-strength concrete in general, various factors, including the degree of packing density or pozzolanic reaction, must be considered along with the relatively small degree of cement hydration [5,6,7].

Silica fume is a key component in the production of high-strength concrete or UHPC. The substituted silica fume serves to enhance the material properties of UHPC by providing both physical and chemical effects. Micro-fine particles of silica fume act as a physical filler that densify the internal structure of the cement matrix and promote the compressive strength of concrete [5,8,9,10,11]. For instance, Cohen et al. noted that if 15% of the cement is replaced by silica fume, approximately 200,000 silica fume particles exist in the cement matrix [12]. These silica fume particles can fill the space between the cement particles, densifying the internal structure of the cement to physically enhance the mechanical properties. Furthermore, silica fume also reacts with the cement hydration product of portlandite (Ca(OH)_2_) and water to additionally form calcium silicate hydrates (C–S–H) [5]. This additional formation of C–S–H-known as the pozzolanic reaction can further make the cement matrix denser, ultimately enhancing the mechanical properties of UHPC [13].

Along with using silica fume, high-temperature curing or steam curing is generally required to facilitate the development of the compressive strength of UHPC [3,14]. Under a high-temperature curing condition, the reaction degree of cement hydration can be accelerated. According to Yamato et al., when concrete is cured at 10 °C, the compressive strength of the concrete cannot be developed enough because of the inactive pozzolanic reaction and low degree of cement hydration [15]. On the other hand, high-temperature curing, such as at 60 °C for 3 days or 90 °C for 2 days, can more effectively facilitate the development of the material properties of UHPC and other types of concrete [3,16].

Compared with cement hydration where there is large amount of water (i.e., high w/c ratios), the chemical reaction of UHPC is very complicated because of the role of silica fume and the dual reactions of cement hydration and pozzolanic reactions within a restrained environment (i.e., limited water availability and limited space availability) and curing under high temperature. Working within this context, the current study tried to elucidate the complex actions through micro- and meso-scale characterizations. A set of experiments of compressive strength using a universal testing machine (UTM), X-ray diffraction (XRD), thermogravimetric analysis (TGA), ^29^Si magic angle spinning nuclear magnetic resonance (MAS NMR), and small-angle X-ray scattering (SAXS) was conducted to investigate the structural changes at different scales.

## 2. Materials and Experimental Details

### 2.1. Sample Preparation

Specimens were prepared with the mix proportions described in Table 1. The mix proportion (Table 1) was designed to investigate the effect of heat on cement hydration and pozzolanic reaction under a low w/c ratio. The substitution ratio of 20 wt.% was determined from the selected dosage of silica fume in our previous studies on UHPC formulation [1,2,3]. In the sample labels, _n and _HT indicate the specified curing age and high-temperature curing, respectively. The oxide compositions of the raw materials used are shown in Table 2. Initially, a UHPC dry premixture was fabricated through the homogeneous blending of ordinary Portland cement (OPC) and silica fume (Grade 940U, Elkem, Norway) using a Hobart mixer for 5 min after which water and a polycarboxylate ether-based superplasticizer (Flowmix 3000U, Dongnam, Korea) were added and mixed together. Then, the fresh mixture was poured into a 50 × 50 × 50 mm^3^ cubic mold. Once the fresh mixture was cast, the surface was immediately covered with a plastic sheet to prevent moisture evaporation, and the specimens were cured for 1 day at 23 °C with a relative humidity (RH) of 60%. Then, the molds were removed, and the sealed specimens were cured using either 23 °C with a RH of 60% or 2 days of high-temperature curing at 90 °C in a water bath (HT specimens in Table 1). After the high-temperature curing, the HT specimens were cured in ambient conditions, 23 °C with a RH of 60% until the subsequent testing at 28 days.

### 2.2. Experimental Details

**Figure 1:**
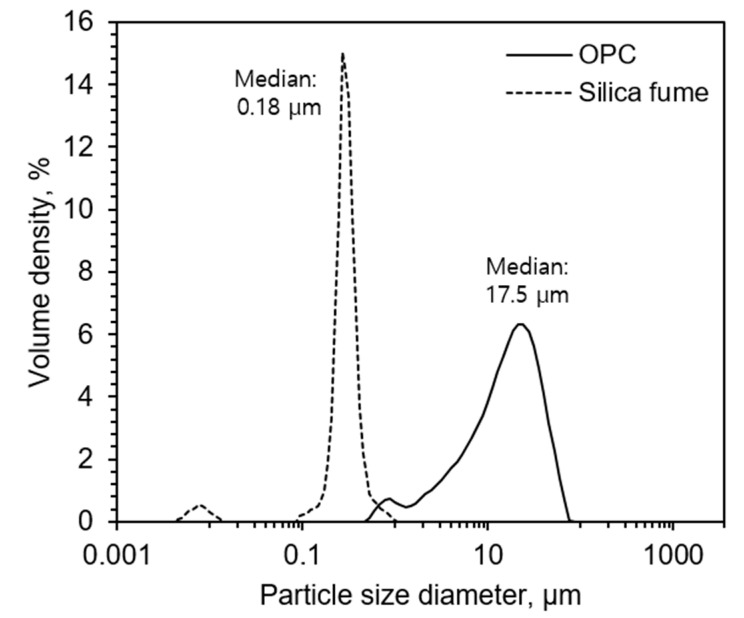
Particle size distribution of cement and silica fume.

The oxide composition and particle size distribution of the powdery form of raw materials were investigated using X-ray fluorescence (XRF) spectroscopy (S4 Pioneer, Bruker AXS GmbH, Germany) and a laser diffraction analyzer (Mastersizer 3000, Malvern Instruments Ltd., Malvern, UK), as shown in Table 2 and Figure 1, respectively. The compressive strength of each paste was determined at 1, 3 and 28 days of curing by taking an average of the three specimens.

The compressive strength test was performed according to ASTM C109 [17]. To determine the strength of each sample, three identical cubes (50 mm × 50 mm × 50 mm) were tested by a universal testing machine. Specimens cured in 1, 3, and 28 days were selected for the compressive strength test. The curing dates were selected to figure out the effect of high temperature curing and the dual-mechanism of pozzolanic reaction and cement hydration.

XRD measurements of the 28 days specimens were performed using an X-ray diffractometer (LabX XRD-6000, Shimadzu Co., Tokyo, Japan) with Cu-Kα radiation (λ = 1.5418 Å). The diffraction patterns of the hardened paste specimens were analyzed using the Crystallography Open Database (COD). To stop the hydration at a certain curing date, specimens were soaked in isopropyl alcohol, washed with diethyl ether, and subsequently dried under 40 °C [18,19,20]. To identify the amount of amorphous phase in the XRD analysis, crystalline TiO_2_ (SRM 674b, NIST, Gaithersburg, MD, USA) was used as an internal standard (TiO_2_: paste = 1:9). Rietveld refinement was applied using TOPAS software and fitted R_wp_ value was around 7. TGA was performed with a DSC/TG system (SDT Q600, TA Instrument Ltd., Newcastle, DE, USA) within a N_2_ environment under a heating rate of 10 K/min up to 1050 °C.

The ^29^Si MAS NMR experiment was conducted using an Advance III HD (Bruker, Karlsruhe, Germany) at 119.182 MHz. The NMR spectra were obtained using a 5 mm HX CPMAS probe and a 5 mm zirconia rotor employing a spinning speed of 10.0 kHz, a pulse width of 2.2 μs, and a relaxation delay of 22 s. ^29^Si chemical shifts were referenced to an external sample of tetrakis (trimethylsilyl) silane at −135.5 ppm with respect to tetramethylsilane (TMS) at 0 ppm and aqueous AlCl_3_ at 0 ppm, respectively.

The SAXS patterns were collected in the *q* range of 0.0014–0.14 nm^−1^ using XEUSS 2.0 equipment (Xenocs, Grenoble, France). The Cu-K*α* X-ray source was operated at 50 kV/0.6 mA with a sample-to-detector distance of 2500 mm. The obtained 2D SAXS data were converted to 1D data by azimuthal integration using Foxtrat 3.4.9 software.

## 3. Experimental Results

### 3.1. Compressive Strength Development

**Figure 2:**
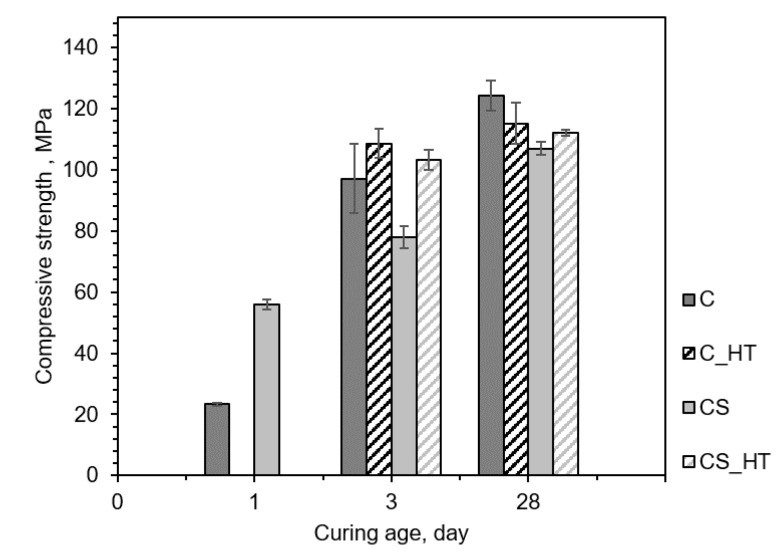
Development of the compressive strength of the specimens.

The development of the compressive strength is presented in Figure 2. The compressive strength of all the samples generally increased as the curing age increased. The CS_1 specimen had a higher early strength than C_1 because of the well-known physical filler effect in which the silica fume became firmly packed in the cement matrix. After 3 days of curing at an ambient temperature, C_3 developed a higher strength than CS_3, indicating that the effect of cement hydration on strength was dominant over the physical filler effect from the silica fume and the strength contribution from the pozzolanic reaction. This could also be explained by the dilution effect (i.e., 20% of the cement was substituted with silica fume in the CS samples).

With the high-temperature curing that took place for 1 to 3 days, silica fume particles were more actively involved in the chemical reaction with portlandite and water, generating additional C–S–H as a secondary reaction. Secondary C–S–H formation efficiently densified the cement matrix and increased compressive strength [6,7,21,22]. Therefore, CS_3_HT showed a higher increased compressive strength during the high-temperature curing. The high-temperature curing also induced the acceleration of the pozzolanic reaction, hence overcoming the dilution effect, which could be confirmed by a similar compressive strength of C_3 and CS_3_HT. This confirmed that an accelerated pozzolanic reaction under a high temperature was more efficient when compared with the acceleration of cement hydration alone.

At 28 days, C_28 showed a slightly higher compressive strength than C_28_HT. During the high-temperature curing, rapidly evaporated or consumed water would tend to have less of an opportunity to fully develop the concrete’s compressive strength, especially at later stages of curing. Therefore, for the two specimens C_28_HT and CS_28_HT, which were cured under a high temperature on days 1 to 3, further strength development was negligible.

### 3.2. XRD and TGA Results

**Figure 3:**
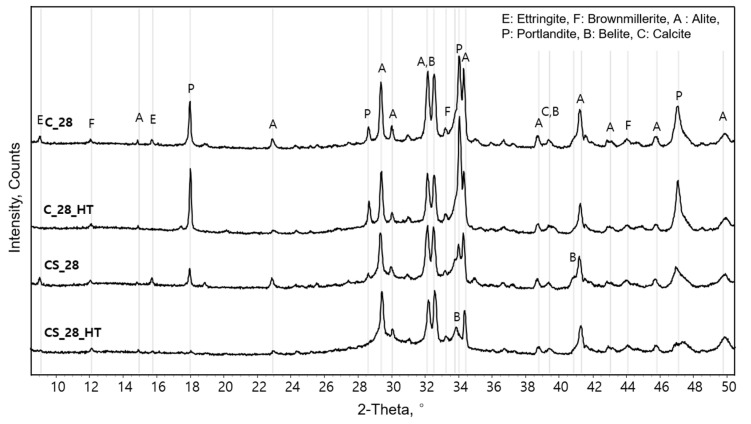
Qualitative XRD analysis patterns of the 28-day specimens.

Figure 3 shows the qualitative XRD analysis results of all the specimens cured at 28 days (C_28, C_28_HT, CS_28, CS_28_HT). The XRD peaks of portlandite were found at 18, 28, 34, and 47°. In general, peaks of portlandite increase and those of clinker minerals decrease under high-temperature curing. Specifically, the CS_28 sample with a silica fume substitution was found to have a smaller amount of portlandite than C_28 and C_28_HT because of the pozzolanic reaction. Furthermore, this pozzolanic reaction (i.e., portlandite consumption reaction) was significantly accelerated under a high temperature, which was confirmed by the full consumption of the produced portlandite in the CS_28_HT specimen [23,24,25].

**Figure 4:**
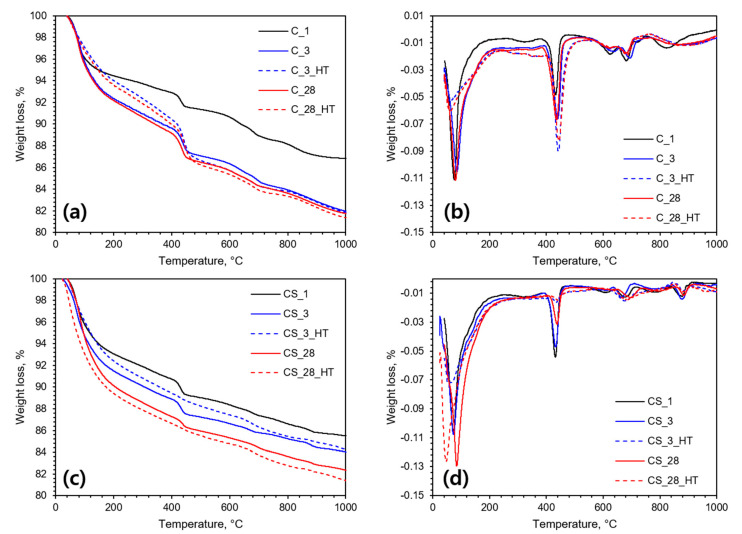
Measured (**a**) TGA of the C specimens, (**b**) derivative TGA of the C specimens, (**c**) TGA of the CS specimens, and (**d**) derivative TGA of the CS specimens.

This phenomenon was also confirmed by the conducted TGA experiment (Figure 4). As the hydration process progresses, an increased amount of chemically bound water was found in the steeper descending curves of total weight loss. Due to the accelerated pozzolanic reaction during the high-temperature curing, a negligible amount of portlandite was found in the CS_3_HT and CS_28_HT specimens. The weight loss around 600 °C had occurred due to the included calcite in raw cement which was also confirmed in LOI (2.2%) from XRF and a consistent amount of calcite refined using XRD data. Therefore, it could be safely assumed that the carbonation was negligible [26].

**Figure 5:**
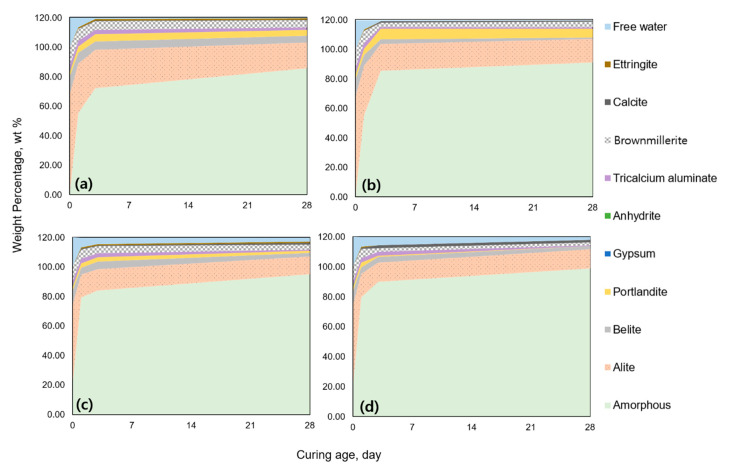
Phase assemblages of all the specimens from the XRD and TGA results: (**a**) C specimen, (**b**) C_HT specimen, (**c**) CS specimen, and (**d**) CS_HT specimen.

By combining the quantitative XRD result using internal standard and the TGA result, phase assemblages of the investigated specimens could be drawn, as shown in Figure 5. All phases that were quantitatively analyzed using the Rietveld method were corrected with the amount of added internal standard sample [27]. Finally, the weight ratio of the phases was obtained considering the amount of chemically bounded water (CBW) analyzed by the TGA results. Because both the C–S–H and silica fume are XRD nondetectable phases, the sum of the two phases was found to be amorphous phase, as shown in Figure 5. Although the amorphous phase possibly included silica fume (i.e., to be discussed in NMR section), the amount of amorphous content had steadily increased in CS and CS_HT because of the additional production of C–S–H from the pozzolanic reaction.

### 3.3. ^29^Si MAS NMR Result

**Figure 6:**
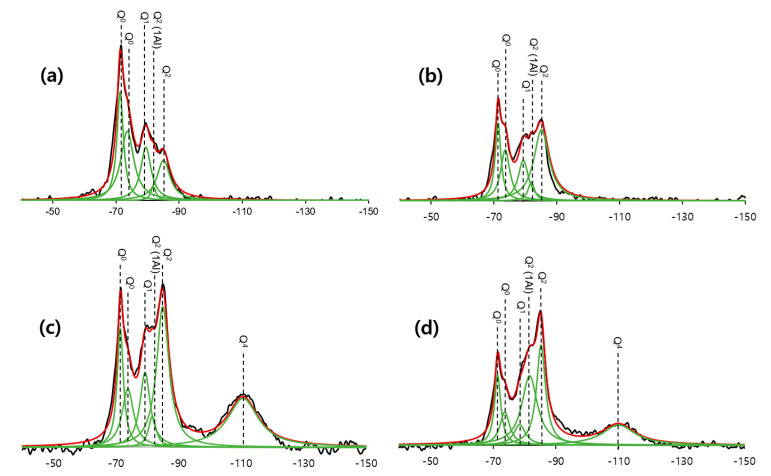
^29^Si magic angle spinning nuclear magnetic resonance **(**^29^Si MAS NMR) spectra and deconvolutions of the 28-day specimens: (**a**) C_28, (**b**) C_28_HT, (**c**) CS_28, (**d**) CS_28_HT)

Figure 6 shows the ^29^Si MAS NMR spectra. The spectra demonstrated narrow resonances at −71, −74, and −79 ppm as the Q^1^ peaks, −81 ppm as the Q^2^(1Al) peak, −85 ppm as the Q^2^ peak, and −110 ppm as the Q^4^ peak. All of the peaks were deconvoluted via Gaussian and Lorentzian functions, and the deconvolution result is presented in Table 3 [26,28]. The specimens cured at high temperatures (C_28_HT and CS_28_HT) showed a decreased Q^0^ peak which indicated the consumption of C_3_S and C_2_S. The resonance at −110 ppm in the CS_28 and CS_28_HT meant the presence of silica fume [29]. The remaining silica fume, even after the accelerated pozzolanic reaction, indicated that the reaction was completed because of the full consumption of water in CS_28 and portlandite in CS_28_HT.

For high-temperature curing, the Q^1^ peak decreased and the Q^2^ peak increased for each sample when compared with the ambient curing condition. The Q^1^ peak appeared at the end of the silicate chain, illustrating the beginning and end of the silicate chain. On the other hand, the Q^2^ peak and Q^2^(1Al) peak constituted the connected silicate chain. Therefore, it could be found that the length of the silicate chain was increased during high-temperature curing. For a more quantitative analysis, the mean chain length (MCL)—the average length of the silicate chain in the silicate material—was calculated using Equation (1) [30,31].

(1) $${\mathrm{MCL}}_{\mathrm{nc}}=\frac{2[Q^{1}+Q^{2}+\frac{3}{2}Q^{2}\left(1\mathrm{Al})\right]}{Q^{1}}$$

The increased rate of MCL with high-temperature curing was found to be larger in CS_28_HT. In CS_28_HT, a secondary C–S–H product was produced by the pozzolanic reaction; therefore, the size of the hydration product of C–S–H should be increased. This phenomenon explains the large increased rate of MCL in CS_28_HT compared with C-28_HT.

Previous studies have shown that aluminum can be substituted for the silicate chain of C–S–H in place of silicon [32,33]. The Q^2^(1Al) peak confirmed the substitution of aluminum in the C–S–H silicate chain, which also increased the intensity value of −81 ppm for Q^2^(1Al) (Table 3). The estimated Al/Si in C–S–H is calculated via Equation (2) [26]. The Al/Si ratio also increased with high-temperature curing and the addition of silica fume. The observed higher Al substitution has been previously reported when it contained silica fume [26].

(2) $$\frac{\mathrm{Al}}{{\mathrm{Si}}_{\mathrm{nc}}}=\frac{\frac{1}{2}Q^{2}\left(1\mathrm{Al}\right)}{Q^{1}+Q^{2}+Q^{2}\left(1\mathrm{Al}\right)}$$

### 3.4. SAXS Results

A SAXS analysis can evaluate the internal structural properties at the meso-scale. A SAXS profile is represented by the Porod region and the fractal region, where the fractal region is divided into a surface fractal section and a mass fractal section [35]. In the current study, an analysis was performed in the fractal section only because the measurement range of the Porod region was narrow.

In the fractal region, the effects of silica fume on the meso-scale structure of cement paste were evaluated based on the fractal model, as suggested by Allen et al. [36]. Fractal-like objects that were structured as packing of colloidal particles were modeled as C–S–H gels. The globules consisted of solid C–S–H layers and an internal water layer [37,38]. The fractal model, described in Equation (3), was composed of a volume fractal scattering term, I_v_(q), and a surface fractal scattering term, *I_s_*(*q*), as described below (Equations (4) and (5)). BGD indicated incoherent flat background scattering.

(3) $$I\left(q\right)=I_{V}\left(q\right)+I_{S}\left(q\right)+\mathrm{BGD}$$

(4) $$I_{V}\left(q\right)=f_{\mathrm{CSH}}V_{P}{\left|\Delta \rho \right|}^{2}F^{2}\left(q\right)[\frac{\eta }{\beta }{\left(\frac{R_{C}}{R_{0}}\right)}^{3}\left(\frac{\xi_{V}}{R_{C}}{)}^{D_{V}}\frac{\sin\left[\left(D_{V}-1\right)\mathrm{atan}\left({q\xi }_{V}\right)\right]}{\left(D_{V}-1\right){q\xi }_{V}{\left(1+{\left({q\xi }_{V}\right)}^{2}\right)}^{(D_{V}-1)/2}}+{\left(1-\eta \right)}^{2}\right]$$

Where $f_{\mathrm{CSH}}$ is the volume fraction of solid C–S–H gel,${\;V}_{P}$ is the particle volume,$\;{\left|\Delta \rho \right|}^{2}$ is the scattering contrast, and $F^{2}\left(q\right)$ is the form factor for spheroidal particles with a size of $R_{0}$. In addition, η is the local packing fraction, β is the aspect ratio of the radii of the spheroid,${\;R}_{C}$ is the basic building block size of the globules (i.e., $R_{C}=2R_{0}$), and $\xi_{V}$ is the upper-limit length scale for the volume fractal.

(5) $$I_{S}\left(q\right)=\pi {\left|\Delta \rho \right|}^{2}\xi_{S}^{4}\frac{S_{0}\Gamma \left(5-D_{S}\right)\sin[\left(3-D_{S})\tan^{-1}\left({q\xi }_{S}\right)\right]}{{q\xi }_{S}{\left(1+{\left({q\xi }_{S}\right)}^{2}\right)}^{(5-D_{S})/2}}$$

where $S_{0}$ is the smooth geometric surface area for the surface fractal microstructure, Γ(x) is the Gamma function, and $\xi_{S}$ is the upper-limit length scale for the surface fractal.

To fit the fractal model systematically to the obtained SAXS spectra, two ranges based on a shoulder peak in the SAXS profile were used to divide the magnitude of the scattering vectors into low and high q ranges because the surface fractal term was associated with a low q range, while the volume fractal term was attributed to a high q range [39]. Then, the coefficients of the volume and surface fractal term were calculated. Both the values of F(q) and β were selected as 1.0 for the calculation [35,36]; $f_{\mathrm{CSH}}V_{P}{\left|\Delta \rho \right|}^{2}$ in the volume fractal term and $S_{0}{\left|\Delta \rho \right|}^{2}$ in the surface fractal term were regarded as constants [40].

**Figure 7:**
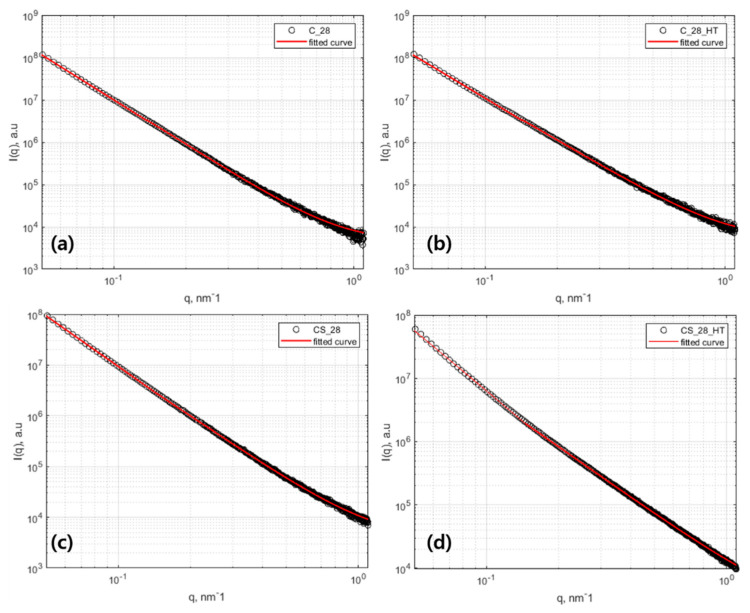
Small-angle X-ray scattering (SAXS) fitting results: (**a**) C_28, (**b**) C_HT_28 (**c**) CS_28, and (**d**) CS_HT_28.

As a result of the SAXS fitting, as shown in Table 4 and Figure 7, the surface fractal dimension (D_S_) increased and the mass fractal dimension (D_V_) decreased in the high-temperature curing setting in all specimens. This higher fractal dimension indicated that the surface of the globule was becoming complicated; however, the internal structure of the globule became more simplified. The amount of cement hydration product increased because of high-temperature curing (especially with secondary C–S–H formation on the exiting C–S–H grain), making the D_s_ of the C–S–H globule complicated in the meso-scale structure. However, because of the high-temperature curing, the pores in the water between the C–S–H layers became smaller and the general pore network to be simpler, decreasing the mass fractal dimension. Because the solubility of cement particles increased during the high-temperature curing, an additional hydration reaction was activated with the remaining unhydrated cement particles under a scenario with limited water and limited space.

**Figure 8:**
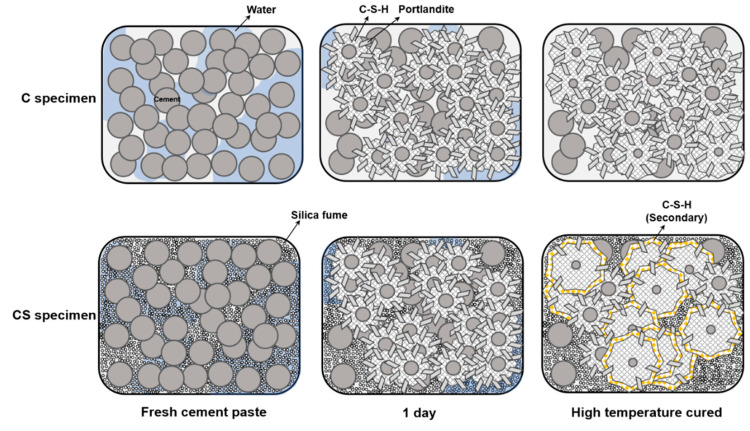
Schematic representation of the cement hydration and pozzolanic reaction under a low w/c ratio.

A decrease of D_v_ demonstrated that multiple but small packing appeared because of the increase of the additional hydration reaction of the residual cement particles. As shown in Figure 2, C_3_HT and CS_3_HT showed only a slight increase in compressive strength when there was an increase of hydration reaction with residual cement particles under high-temperature curing. Therefore, with this dual mechanism, because the water was already consumed in the early curing stage, cement hydration enhancement, including the crystal growth mechanism, did not occur. It resulted in a compressive strength after 28 days that was almost constant. Regarding C_28_HT and CS_28_HT, as shown in Figure 2, an increase in the long-term compressive strength showed less of an increase than in the early curing stage because of the pre-consumption of water; this result showed that the D_v_ and compressive strength could be related to be proportional to each other [41]. With the substitution of silica fume, the value of R_c_ was significantly increased compared with the C_28 and C_28_HT specimens because of the secondary hydration product forming around the existing cement hydrate. This explanation was graphically illustrated further in Figure 8.

## 4. Discussion

### 4.1. Cement Hydration Under Low Water Amount and High Temperature

The cement hydration reaction was more active under high-temperature curing. As demonstrated in Figure 2, C_3_HT exhibited a higher compressive strength than C_3. Specimens placed under high-temperature curing had a greater compressive strength because of higher dissolution of cement particles and subsequent faster cement hydration leading to the production of more of cement hydrates. The results of the XRD and TGA quantitatively confirmed the accelerated pozzolanic reaction which could well be explained by the readily available water and portlandite around the cement hydrates.

The observed intensities of clinker minerals (especially C_3_S and C_2_S) decreased under high temperature curing. Focusing on the change in the silicate chain in C–S–H as analyzed by ^29^Si MAS NMR, the resonant intensity of the Q^0^ peak decreased in both C_28_HT and CS_28_HT under high-temperature curing. It also supported more hydration as the Q^0^ peak belonged to the C_3_S and C_2_S. The intensity of the Q^1^ peak decreased and the intensity of the Q^2^ peak increased, resulting in an increase in the value of MCL. Through this, the activation of a cement hydration reaction could be seen as making the average length of the silicate chain longer [42,43,44].

To examine the structural changes at the meso-scale, SAXS was performed to analyze the C–S–H globules by their fractal dimension [39]. As the cement paste cured at high temperatures, the amount of cement hydration products increased. Thus, D_s_ increased but as the pore size decreased and the pore structure simplified, the D_v_ of the specimen decreased. Therefore, when performing high-temperature curing on cement with a low w/c ratio, it was observed that the size of the globules increased and the surface became complicated, but the internal pore structure became untangled.

### 4.2. Pozzolanic Reaction Under a Low Amount of Water and High Temperature

The pozzolanic reaction is a secondary reaction that consumes silica fume, water, and the hydration product of portlandite. The experimental results conducted herein revealed the structural modification from the secondary reaction under high temperature. Figure 8 shows the schematic view of the secondary reaction with silica fume. As the solubility of amorphous SiO_2_ stayed similar under 100 °C, the pozzolanic reaction could be less affected by the increased solubility of the silica fume [45]. On the other hand, the silica fume could easily react with the preproduced portlandite and water to enlarge the hydration product colored with yellow in Figure 8. Comparing the compressive strength of CS_3_HT with silica fume to be activated for the pozzolanic reaction, an increased rate of the compressive strength was more prominent than C_3_HT without the pozzolanic reaction. As demonstrated in Figure 2, CS_3_HT showed a 32% higher compressive strength than CS_3. This is a significantly larger value than the increasing rate relation with C_3_HT and C_3, which showed a 12% compressive strength enhancement [46]. Therefore, when performing high-temperature curing, the pozzolanic reaction acts more dominantly than the cement hydration reaction. When comparing CS_28 and CS_28_HT in the qualitative and quantitative XRD results (Figure 5 and Figure 8), the amount of portlandite generated was less than C_28 and C_28_HT because of pozzolanic reaction as well as the dilution effect (i.e., the CS series contained less cement). Moreover, portlandite was hardly found in CS_28_HT, indicating that the pozzolanic reaction was terminated by the full consumption of portlandite. Meanwhile, in the CS_28 sample, there were remaining silica fume and portlandite so it can be inferred that the pozzolanic reaction had not been completed by the third day of curing. The subsequent compressive strength development from 3 days to 28 days supports the continuous but not terminated pozzolanic reaction in ambient curing conditions.

As shown in Figure 6, ^29^Si MAS NMR indicated that the Q^4^ peak was significantly reduced after high-temperature curing. Further, when looking at the intensity of the spectra, CS_28 and CS_28_HT had a smaller Q^0^ peak than C_28 and C_28_HT, which also proved that the amount of clinker minerals had a different intensity of resonance because of the small amount of absolute cement in the CS mix proportion. In CS_28_HT, a similar tendency as in the C_28_HT showed that the MCL became longer when subjected to high-temperature curing. However, the increased rate of the MCL in CS_28_HT was greater than the increased rate in C_28_HT. The secondary pozzolanic reaction had more influence on the MCL of C–S–H than the cement hydration reaction [31]. Similarly, in the meso-scaled analysis, the R_c_ value measured with SAXS was greatly increased as it contained silica fume. These meso-scale characteristics could also be explained by the additional formation of C–S–H on the grain of existing C–S–H. Because the pozzolanic reaction should consume the hydration product of portlandite and does not require the anhydrous cement, the reaction can be more easily accelerated under high temperature conditions because it does not rely on the diffusion of water through the cement hydrate to the anhydrous particles.

## 5. Conclusions

In the current study, micro- and meso-structural changes of cement pastes under a low w/c ratio environment, heat treatment, the presence of silica fume were investigated through various experiments (i.e., compressive strength test, XRD, TGA, ^29^Si MAS NMR, SAXS). The conclusions of the current study are given below.

1. In the initial curing stage, the silica-fume-added specimen showed a greater compressive strength because of the physical filler effect of silica fume. For 3 days of curing, cement hydration showed to be more dominant without high-temperature curing. With high-temperature curing, the pozzolanic reaction was more easily accelerated in enhancing the compressive strength. However, with 28 days of curing, the high-temperature-cured specimens showed negligible strength development because it diminished the opportunity of increasing the long-term compressive strength through rapid consumption of the given low amount of water.
2. Quantitative and qualitative XRD analyses confirmed that high-temperature curing showed higher portlandite formation than at ambient temperature curing. High-temperature curing produces more portlandite crystals because high temperature enhanced the reactivity of clinker minerals. On the other hand, when silica fume was substituted with 20% of cement, the pozzolanic reaction was more easily accelerated compared to the acceleration degree of cement hydration. Negligible amounts of portlandite were found after the high temperature curing as a result of pozzolanic reaction. The reason was suggested by the readily accessible ingredients of the pozzolanic reaction (i.e., silica fume, water, portlandite) in a restrained environment and less dependency of water diffusion for activating the pozzolanic reaction. This suggestion was reinforced by the result of SAXS analysis.
3. The ^29^Si MAS NMR experiment was performed to investigate the silicate chain of C–S–H. High-temperature curing resulted in an increase of the Q^2^ peaks, which is the inner part of the silicate chain, and a decrease of the Q^1^ peak, which is the edge peak. As silica fume particles participated in the pozzolanic reaction and in forming the additional C–S–H on the existing C–S–H particles, the increased rate of MCL under high-temperature curing was larger than in the non-added specimens. The existence of Q^4^ peak at 28 days indicated that the pozzolanic reaction was terminated due to the full consumption of water or portlandite during the heat treatment.
4. Structural changes in the meso-scale were investigated with SAXS. With a high-temperature curing, the fractal dimension increased with enhanced cement hydration. The higher reaction degree of the clinker minerals made the surface of the C–S–H globule complicated and enlarged the size of the globule. On the other hand, because of the consumption of water by rapid hydration reaction under heat treatment, the internal structure of the composite became simpler, making the mass fractal dimension smaller. The pozzolanic reaction led to higher mass and surface fractal values, indicating that the pozzolanic reaction can be more easily accelerated under high temperature curing conditions. This can be explained by the fact that the pozzolanic reaction does not require the anhydrous as a reaction precursor. It consumes the hydration product of portlandite which will be more readily available on the surface of preproduced C–S–H where more water should be available for subsequent reactions. Therefore, the reaction does not require the diffusion mechanism of water for more crystal growth or further hydration. Therefore, the effect of the acceleration of the pozzolanic reaction under high temperature is more dominant in the low w/c environment.

## Figures and Tables

**Table 1:** Mix proportions of specimens by weight percentage of cement, wt.%.

Mixture	Cement	Silica Fume	Water	Super-Plasticizer
C_n	100	0	20	4
C_n_HT *	100	0	20	4
CS_n	80	20	20	4
CS_n_HT	80	20	20	4

* HT indicates heat treatment

**Table 2:** Oxide compositions of ordinary Portland cement (OPC) and silica fume, wt.%.

Ordinary Portland Cement				Silica Fume			
Formula	Concentration, %	Formula	Concentration, %	Formula	Concentration, %	Formula	Concentration, %
CaO	62.9	K_2_O	0.37	CaO	1.54	K_2_O	0.64
SiO_2_	21.00	Na_2_O	0.29	SiO_2_	96.90	Na_2_O	0.16
Al_2_O_3_	4.15	P_2_O5	0.18	Al_2_O_3_	0.29	P_2_O5	0.05
MgO	3.13	SrO	0.10	MgO	0.18	SrO	-
Fe_2_O_3_	2.93	MnO	0.06	Fe_2_O_3_	0.15	MnO	0.03
LOI *	2.20	ZnO	0.04	LOI	0.02	ZnO	-
SO_3_	2.15	CuO	0.04	SO_3_	-	CuO	-
TiO_2_	0.41	NiO	0.02	TiO_2_	0.01	NiO	-
Total	99.97	-	-	Total	99.97	-	-

* Loss of ignition.

**Table 3:** Deconvolution results for ^29^Si MAS NMR spectra, wt.% [34].

Specimen	Q^0^ −71 ppm	Q^0^ −74 ppm	Q^1^ −79 ppm	Q^2^(1Al) −81 ppm	Q^2^ −85 ppm	Q^4^ −110 ppm	Al/Si	MCL
C_28	13.56	8.66	6.67	1.73	5.03	0	0.064	4.29
C_28_HT	10.00	6.54	5.23	2.40	9.16	0	0.071	6.88
CS_28	11.41	5.60	7.17	2.96	13.49	4.70	0.063	7.00
CS_28_HT	9.93	4.69	2.92	9.85	14.18	2.87	0.183	21.80

**Table 4:** Volume and surface fractal parameters derived from SAXS fitting of the fractal model.

Sample	Volume Fractal Parameters					Surface Fractal Parameters		
	R_c_ (nm)	D_v_	ξ_v_ (nm)	η	f_C–S–H_V_p_|Δρ|^2^ (nm)	D_S_	ξ_S_ (nm)	S_0_|Δρ|^2^ (nm)
C_28	8.58	2.91	29.70	1.25	19.61	2.46	1385.8	3.2 × 10^−4^
C_28_HT	11.89	2.84	28.64	1.27	19.40	2.59	668.86	3.2 × 10^−4^
CS_28	23.21	2.77	20.49	1.92	13.57	2.66	926.98	3.1 × 10^−4^
CS__28_HT	50.06	2.52	17.52	1.45	12.22	2.80	305.93	3.9 × 10^−4^

## References

[1] Kang S.-H., Hong S.-G., Moon J. (2019). The use of rice husk ash as reactive filler in ultra-high performance concrete. Cem. Concr. Res..

[2] Kang S.-H., Jeong Y., Tan K.H., Moon J. (2018). The use of limestone to replace physical filler of quartz powder in UHPFRC. Cem. Concr. Comp..

[3] Kang S.-H., Lee J.-H., Hong S.-G., Moon J. (2017). Microstructural Investigation of Heat-Treated Ultra-High Performance Concrete for Optimum Production. Materials.

[4] Teichmann T., Schmidt M. Influence of the packing density of fine particles on structure, strength and durability of UHPC. Proceedings of the International Symposium on Ultra High Performance Concrete.

[5] Oertel T., Helbig U., Hutter F., Kletti H., Sextl G. (2014). Influence of amorphous silica on the hydration in ultra-high performance concrete. Cem. Concr. Res..

[6] Weng J.K., Langan B., Ward M. (1997). Pozzolanic reaction in Portland cement, silica fume, and fly ash mixtures. Can. J. Civ. Eng..

[7] Yogendran V., Langan B.W., Ward M.A. (1991). Hydration of cement and silica fume paste. Cem. Concr. Res..

[8] Soroka I., Setter N. (1977). The effect of fillers on strength of cement mortars. Cem. Concr. Res..

[9] Shih J.-Y., Chang T.-P., Hsiao T.-C. (2006). Effect of nanosilica on characterization of Portland cement composite. Mater. Sci. Eng. A.

[10] Shannag M.J. (2000). High strength concrete containing natural pozzolan and silica fume. Cem. Concr. Comp..

[11] Yogendran V., Langan B.W., Haque M.N., Ward M.A. (1987). Silica Fume in High-Strength Concrete. ACI Mater. J..

[12] Cohen M.D., Olek J., Dolch W.L. (1990). Mechanism of plastic shrinkage cracking in portland cement and portland cement-silica fume paste and mortar. Cem. Concr. Res..

[13] Land G., Stephan D. (2012). The influence of nano-silica on the hydration of ordinary Portland cement. J. Mater. Sci..

[14] Shen P., Lu L., He Y., Rao M., Fu Z., Wang F., Hu S. (2018). Experimental investigation on the autogenous shrinkage of steam cured ultra-high performance concrete. Constr. Build. Mater..

[15] Yamato T., Emoto Y., Soeda M. (1986). Strength and freezing-and-thawing resistance of concrete incorporating condensed silica fume. Spec. Publ..

[16] Kwon Y.-H., Kang S.-H., Hong S.-G., Moon J. (2017). Intensified Pozzolanic Reaction on Kaolinite Clay-Based Mortar. Appl. Sci..

[17] Astm A. (2013). Standard test method for compressive strength of hydraulic cement mortars (using 2-in or [50-mm] cube specimens). Ann. Book ASTM Stand..

[18] Zhang J., Scherer G.W. (2011). Comparison of methods for arresting hydration of cement. Cem. Concr. Res..

[19] Snellings R., Chwast J., Cizer Ö., De Belie N., Dhandapani Y., Durdzinski P., Elsen J., Haufe J., Hooton D., Patapy C. (2018). Report of TC 238-SCM: Hydration stoppage methods for phase assemblage studies of blended cements—results of a round robin test. Mater. Struct..

[20] De La Torre A., Bruque S., Aranda M. (2001). Rietveld quantitative amorphous content analysis. J. Appl. Crystallogr..

[21] Zhang Z., Zhang B., Yan P. (2016). Hydration and microstructures of concrete containing raw or densified silica fume at different curing temperatures. Constr. Build. Mater..

[22] Zhang M.-H., Gjørv O.E. (1991). Effect of silica fume on cement hydration in low porosity cement pastes. Cem. Concr. Res..

[23] Schachinger I., Hilbig H., Stengel T., Fehling E. Effect of curing temperature at an early age on the long-term strength development of UHPC. Proceedings of the 2nd International Symposium on Ultra High Performance Concrete.

[24] Heinz D., Urbonas L., Gerlicher T. Effect of heat treatment method on the properties of UHPC. Proceedings of the Hipermat 2012 3rd International Symposium on UHPC and Nanotechnology for High Performance Construction Materials.

[25] Cheng-Yi H., Feldman R.F. (1985). Influence of silica fume on the microstructural development in cement mortars. Cem. Concr. Res..

[26] Lee N.K., Koh K.T., Kim M.O., Ryu G.S. (2018). Uncovering the role of micro silica in hydration of ultra-high performance concrete (UHPC). Cem. Concr. Res..

[27] Jeong Y., Hargis C., Kang H., Chun S.-C., Moon J. (2019). The Effect of Elevated Curing Temperatures on High Ye’elimite Calcium Sulfoaluminate Cement Mortars. Materials.

[28] Metz K.R., Lam M.M., Webb A.G. (2000). Reference deconvolution: A simple and effective method for resolution enhancement in nuclear magnetic resonance spectroscopy. Concept. Magn. Reson..

[29] Pena P., Rivas Mercury J.M., de Aza A.H., Turrillas X., Sobrados I., Sanz J. (2008). Solid-state 27Al and 29Si NMR characterization of hydrates formed in calcium aluminate–silica fume mixtures. J. Solid State Chem..

[30] Richardson I., Groves G. (1993). The incorporation of minor and trace elements into calcium silicate hydrate (C–S–H) gel in hardened cement pastes. Cem. Concr. Res..

[31] Wang J., Han B., Li Z., Yu X., Dong X. (2019). Effect Investigation of Nanofillers on C–S–H Gel Structure with Si NMR. J. Mater. Civ. Eng..

[32] Andersen M.D., Jakobsen H.J., Skibsted J. (2004). Characterization of white Portland cement hydration and the C–S–H structure in the presence of sodium aluminate by 27Al and 29Si MAS NMR spectroscopy. Cem. Concr. Res..

[33] Thomas J.J., Rothstein D., Jennings H.M., Christensen B.J. (2003). Effect of hydration temperature on the solubility behavior of Ca-, S-, Al-, and Si-bearing solid phases in Portland cement pastes. Cem. Concr. Res..

[34] Martínez-Ramírez S., Frías M. (2009). The effect of curing temperature on white cement hydration. Constr. Build. Mater..

[35] Allen A.J., Oberthur R.C., Pearson D., Schofield P., Wilding C.R. (1987). Development of the fine porosity and gel structure of hydrating cement systems. Philos. Mag. B.

[36] Allen A.J., Thomas J.J., Jennings H.M. (2007). Composition and density of nanoscale calcium–silicate–hydrate in cement. Nat. Mater..

[37] Jennings H.M. (2008). Refinements to colloid model of C–S–H in cement: CM-II. Cem. Concr. Res..

[38] Chiang W.-S., Fratini E., Baglioni P., Liu D., Chen S.-H. (2012). Microstructure Determination of Calcium-Silicate-Hydrate Globules by Small-Angle Neutron Scattering. J. Phys. Chem. C.

[39] Allen A.J., Thomas J.J. (2007). Analysis of C–S–H gel and cement paste by small-angle neutron scattering. Cem. Concr. Res..

[40] Vollet D.R., de Sousa W.A.T., Donatti D.A., Ibañez Ruiz A. (2007). Mass fractal characteristics of sonogels prepared from sonohydrolysis of tetraethoxysilane with additions of dimethylformamide. J. Non-Cryst. Solids.

[41] Jin S., Zhang J., Chen C.Z., Chen W.L. (2011). Study of pore fractal characteristic of cement mortar. Jianzhu Cailiao Xuebao/J. Build. Mater..

[42] Al-Dulaijan S.U., Parry-Jones G., Al-Tayyib A.-H.J., Al-Mana A.I. (1990). 29Si Magic-Angle-Spinning Nuclear Magnetic Resonance Study of Hydrated Cement Paste and Mortar. J. Am. Ceram. Soc..

[43] Parry-Jones G., Al-Tayyib A.J., Al-Dulaijan S.U., Al-Mana A.I. (1989). 29Si MAS-NMR hydration and compressive strength study in cement paste. Cem. Concr. Res..

[44] Sáez del Bosque I.F., Martín-Pastor M., Martínez-Ramírez S., Blanco-Varela M.T. (2013). Effect of Temperature on C3S and C3S + Nanosilica Hydration and C–S–H Structure. J. Am. Ceram. Soc..

[45] Fournier R.O., Rowe J.J. (1977). The solubility of amorphous silica in water at high temperatures and high pressures. Am. Mineral..

[46] Rong Z.D., Sun W., Xiao H.J., Wang W. (2014). Effect of silica fume and fly ash on hydration and microstructure evolution of cement based composites at low water–binder ratios. Constr. Build. Mater..

